# HIV-1 genetic diversity and reverse transcriptase resistance mutations in Benin before dolutegravir era, West Africa

**DOI:** 10.1371/journal.pone.0348800

**Published:** 2026-05-21

**Authors:** Edmond Tchiakpe, René Kpemahouton Keke, Patricia Recordon-Pinson, Abou Abdallah Malick Diouara, Marie-Line Andreola, Moussa Bachabi, Aldric Afangnihoun, Eric Gbaguidi, Sophia Osawe, Almoustapha Issiaka Maiga, Halimatou Diop-Ndiaye, Coumba Touré-Kane, Akadiri Yessoufou

**Affiliations:** 1 Laboratory of Cell Biology and Physiology, Department of Biochemistry and Cellular Biology, Faculty of Sciences and Technology (FAST) and Institute of Applied Biomedical Sciences (ISBA), University of Abomey-Calavi, Cotonou, Benin; 2 National Reference Laboratory of Health Program Fighting Against AIDS in Benin (LNR/PSLS), Health Ministry of Benin, Cotonou, Benin; 3 Health Program Fighting Against AIDS in Benin, Cotonou, Benin; 4 UMR5234 MFP, University of Bordeaux and Virology Laboratory, WHO HIV Center, CHU Bordeaux, France; 5 Groupe de Recherche Biotechnologies Appliquées & Bioprocédés Environnementaux (GRBA-BE), Laboratoire Eau-Énergie-Environnement-Procédés-Industriels (LE3PI), École Supérieure Polytechnique (ESP), Université Cheikh Anta Diop, Dakar, Sénégal; 6 Institute of Human Virology, Nigeria, Plateau State, Abuja, Nigeria; 7 Department of Medical Biology, CHU Gabriel Toure, University of Sciences Techniques and Technologies of Bamako, Bamako, Mali; 8 Laboratoire de Bactériologie Virologie CHU Aristide le Dantec, Université Cheikh Anta Diop, Dakar, Sénégal; 9 Institut de Recherche en Santé, de Surveillance Épidémiologique et de Formation (IRESSEF), Pôle Urbain Diamniadio, Dakar, Sénégal; MRC/UVRI & LSHTM Uganda Research Unit, UGANDA

## Abstract

Benin adopted the World Health Organization’s (WHO) “Test and Treat” recommendation in 2016 and, since 2019, has updated this protocol by including dolutegravir (DTG) as the preferred first-line treatment. Study aimed to assess the prevalence of virological failure (VL > 3log copies/mL) and review genetic diversity and antiretroviral resistance mutations in patients on antiretroviral treatment for at least 12 months before dolutegravir Era in Benin. Cross-sectional study included patient plasmas from antiretroviral treatment sites nationwide. Viral load was performed in National Reference Laboratory of Health Program Fighting Against AIDS using the Cobas® 5800 HIV-1 assay. For plasmas those VL above 1000 copies/mL, nested PCR were done along the entire protease and part of reverse transcriptase. The DNA obtained by the Sanger method was used to determine the subtypes of HIV-1 after editing with DNASTAR SeqMan Pro™ and alignment with ClustalW2 version 2.1. Phylogenetic trees were constructed by the Neighbor-Joining method and recombinants were investigated by bootscanning with Seaview software version 2.1. DNA was subjected to the Stanford University Antiretroviral Resistance Mutation Interpretation Algorithm (https://hivdb.stanford.edu/) to identify the positions of drug-associated resistance mutations. (178/253; 70.35%) of the samples were correctly amplified and sequenced. CRF02_AG (n = 104) was the predominant strain observed followed by CRF06_cpx (n = 21), G (n = 5), CRF43_02G (n = 2), CRF37_cpx (n = 2), A1 (n = 1) and unique recombinant forms (URFs) (n = 34) among the 169 samples sequenced on entire protease combined with part of the reverse transcriptase. (164/178; 92.1%), (141/178; 79.2%), (160/178; 89.9%) and (7/169; 4.1%) patients carried at least one drug resistance associated to NRTIs, NNRTIs and PIs respectively. M184I/V, TAMSI: (M41L, L210W, T215Y) and TAMSII: (D67N, K70R, K219Q/E, T215I/V) represented (71.3%), (21.9%), (35.4%) respectively. K103N/S was the most preponderant mutations encountered with a proportion of 64.6% followed by V179E (21.3%), P225H (20.2%), V108I (19.1%), Y181C (16.3%), A98G (15.2%), V106I/M/A (10.1%). I84V (1.8%) mutation was the major associated PIs encountered followed by L90M, V82A, M46I each encountered twice (1.2%) and I47A, V32I, I54V, G48A, each encountered once (0.6%). Study shows a high genetic diversity with the presence of new strains and underlines the need to regularly review data on genetic diversity and resistance among patients receiving antiretroviral therapy in the country.

## Introduction

Benin is a West African country with a concentrated epidemic of HIV-1 infection. HIV-1 prevalence is low in the general population (0.8%) [1,2] and high in key populations such as female sex workers (FSW) and men who have sex with men (MSM), 7.2% and 7.7% % respectively [2]. Management of people living with HIV (PLHIV) began in 2002 with the adoption of « Test and Treat » in 2016. Systematic monitoring of patients on treatment was ensured by viral load quantification and CD4 + T- cell count. Following two consecutive viral loads exceeding 1000 copies/mL at three-month intervals, the patient was declared to have failed treatment [3,4] and resistance genotyping was requested. At the end of 2024, the number of PLHIV was estimated at 70000 and 60000 were actually on antiretroviral treatment, including 57480 adults and 2520 children [5]. For the triple objective 95-95-95 in HIV testing, treatment, and viral suppression, beyond the difficulties encountered, the country reached 89-98-82 in 2023 [6] and 89-99-91 in 2024 [5]. One of the areas on which we must act appears to be the first-line therapeutic line following the study on the primary resistance of HIV-1 in Benin that showed a prevalence of resistance associated with non-nucleoside reverse transcriptase inhibitors (NNRTIs) of around 10% [7]. Likewise, the study assessing resistance to pretreatment integrase inhibitors showed a prevalence of zero percent [8]. These data reinforce Benin’s strategy to adopt the 2019 WHO recommendations to close the gaps and achieve UNAIDS target 95. In children for whom antiretroviral therapy is known to be problematic, especially due to the need for a third party to administer antiretrovirals and monitor treatment adherence, a recent study in the country reported that (25/32; 78.12%), (28/32; 87.5%), (4/32; 12.90%), (22/32; 68.75%), (02/32; 6.25%) of children had at least one drug resistance mutation associated with NRTIs, NNRTIs, PIs, NNRTIs + NRTIs, and NNRTIs + NRTIs + PIs respectively. In addition, the majority of children were on an (efavirenz) EFV-based regimen (22/47; 46.8%). It then becomes clear that these mutations could be the cause of some cases of treatment failure, thus contributing to slowing down the indicators towards reaching the third UNAIDS 95 target. Although proportions of resistance mutations have been reported under therapeutic regimens without dolutegravir at one year [9] two years [10], five years [11], no study has presented the situation of drug resistance mutation in Benin after a twelve-month follow-up.

Thus, before replacing efavirenz by dolutegravir as first-line treatment according to WHO recommendations, a general evaluation of the efficacy of antiretroviral treatment was necessary and having data for the country was crucial. The objective of the study was to assess the prevalence of virological failures and to examine genetic diversity and antiretroviral resistance mutations in patients who had received at least 12 months of antiretroviral treatment before DTG era in Benin.

## Materials and methods

### Study area and population

The cross-sectional study included whole blood samples from adult patients in 22 care centers for people living with HIV-1 (PLHIV-1) nationwide and collected during the period from July 18, 2022 to August 13, 2022. These were the sites that had a number of patients exceeding 1.0% of the total national number of patients in 2021. These patients were on different therapeutic regimens without dolutegravir for at least 12 months of follow-up (inclusion criteria). Patients receiving dolutegravir treatment and children under 18 years of age were excluded from the study population (Exclusion criteria).

The study was authorized by the PSLS in collaboration with the Ministry of Health in Benin and the National Ethics Committee for Health Research (CNERS). Written informed consent was obtained from each study participant. The heads of the decentralized HIV/AIDS care sites also gave their agreement in principle for the implementation of the study.

### Viral load quantification and RNA extraction

Viral load (VL) was determined in National Reference Laboratory of Health Program Fighting Against AIDS (LNR/PSLS) using the Cobas® 5800 HIV-1 assay with a linear range from 20 copies/mL to 1.0 x 10^**7**^ copies/mL for a 500 µL plasma sample [12].

Based on the revised WHO recommendations of 2013 [3,4], any plasma sample with a viral load greater than 3 log copies/mL was included in the analysis and viral RNA was extracted from 140 µl of plasma using the Qiagen kit according to the manufacturer’s instructions in LNR/PSLS and used for RT-PCR and nested PCR along the entire protease (PR) and part of reverse transcriptase (RT).

### RT PCR and Nested PCR amplification on entire protease and partial reverse transcriptase amplification

RT-PCR and nested PCR were performed at the LNR/PSLS on full amplification of the protease and part of the reverse transcriptase using the commercial HIV-1 PR/RT genotyping kit with Integrase from Applied Biosystems™. RT-PCR was done for each reaction in 50 ul composed of 39 ul of RT-PCR Master Mix PR/RT, 1 ul of SuperScript™ III One-Step RT-PCR with Platinum™ Taq High Fidelity enzyme and 10 ul of RNA. Temperatures cycle was composed to one cycle 50°C for 45 min, one cycle 94°C for 2 min, 40 cycles composed of whole 94°C for 15 s, 50°C for 20 s, 72°C for 120s, one cycle of 72°C for 10 min for final extension and for hold at 4°C for maximum 18 hours. Nested PCR was done for each reaction in 50 ul composed of 47.5 ul of nested PCR Master Mix PR/RT, 0.5 ul of AmpliTaq Gold™ LD DNA Polymerase and 2 ul of RT‑PCR product. Temperatures cycle was composed to one cycle 94°C for 4 min, 40 cycles composed of whole 94°C for 15 s, 53°C for 20 s, 72°C for 120s, one cycle of 72°C for 10 min for final extension and for hold at 4°C for maximum 18 hours (https://www.thermofisher.com/order/catalog/product/A55120)

### PCR products purification

PCR products were purified with QIAquick® PCR Purification Kit [13] according to the manufacturer’s instructions. To 50 µl of PCR product, 250 µl of Qiagen PB (purification binding) solution and 10 µl of sodium acetate were added and mixed. The mixture was loaded into a Qiagen column and centrifuged at 1300 rpm for one minute. The DNA was washed with 730 µl of PE (Pre-Elution) solution previously reconstituted with 95% pure ethanol. After centrifugation and complete evaporation of the alcohol, the DNA was recovered in 30–50 µl using Qiagen EB (elution buffer) solution.

### Sequence reactions

Sequencing reactions were performed in LNR/PSLS. The purified DNA (2 ul) was mixed with 18 ul of each of six PR/RT gene sequencing primer mixes F1, F2, F3, R1, R2 and R3. Temperatures cycle was 25 cycles of whole 96°C for 10 s, 50°C for 5 s, 60°C for 4 min and for hold at 4°C for maximum 18 hours (https://www.thermofisher.com/order/catalog/product/A55120).

### DNA precipitation and nucleotide sequencing by Sanger

The DNA obtained was cold precipitated. A mixture of sodium acetate (4 μl) and pure ethanol (40 μl) were mixed with the sequences then vortexed and then deposited at −20°C for at least 30 min. They were centrifuged at 3455 rpm at 4°C for 20 min then removed from the supernatant and resuspended in 150 μl of 95% pure ethanol refrigerated at 4°C then centrifuged at 3455 rpm at 4°C for 20 min. After removal of the supernatant, the ethanol was completely evaporated from the DNA in open air and in the dark and then resuspended in 10 μl of formamide. They were then subjected to Applied Biosystems 3500 series genetic analyzers for DNA sequencing.

### Sequences correction and genetic diversity of the gene covering the entire protease and part of the reverse transcriptase of HIV-1

DNA sequences were edited with DNASTAR SeqMan Pro^TM^ and the FASTA of each region (PR, RT and PR-RT) were assembled in order to determine the HIV-1 genotype. Recombinants were investigated by bootscanning with Seaview software version 2.1. The derived nucleotide sequences of PR, RT and PR-RT sequences was aligned by the ClustalW2 version 2.1 alignment program with known reference strains of M, N, and O pooled from the HIV-1 gene databank (https://www.hiv.lanl.gov/). Phylogenetic trees were inferred using the neighbor-joining method from matrix distances calculated after gap stripping alignments, according to a Kimura two-parameter algorithm. Bootstrapping was performed with 100 replicates. The circular tree was obtained using ITOL website (available online: http://itol.embl.de/, Letunic and Bork (2024) Nucleic Acids Res https://doi.org/10.1093/nar/gkae268).

### Identification of mutation positions along the gene covering the entire protease and part of the reverse transcriptase

DNA were directly submitted to the Stanford University Antiretroviral-Associated Resistance Mutation Interpretation Algorithm (https://hivdb.stanford.edu/) to identify the positions of drug-associated resistance mutations.

The new sequences generated as part of this study have been deposited in Genbank under the following NCBI accession numbers: PV382981 to PV383164.

### Ethics statement

Ethical authorization was obtained from the National Health Research Ethics Committee under number 56 dated December 2, 2021, authorizing the implementation of the study. Written informed consent was obtained from each study participant. The confidentiality and anonymity of each participant’s information were preserved. The study was conducted in accordance with applicable guidelines and regulations.

## Results

### Study population

523 patients were included in the study and 48.37% (253/523) were in virological failure (VL > 3log). For these patients, HIV RNA was correctly amplified and sequenced resulting in a sequencing success proportion of 70.35% (178/253). The median age was 35 years [IQR: 19–71] with 68% women and 32% men. The median viral load was 4.79log_10_ [IQR: 3.31–6.43log_10_]. They were under the therapeutic lines TDF/ABC + 3TC + EFV/ATVr/LPVr (141/178; 79.20%) and AZT/D4T/ + 3TC + NVP/EFV (37/178; 20.80%).

### Distribution of HIV-1 strains along the entire protease gene and part of the reverse transcriptase gene

Genetic diversity from the Pol Gene (PR + part of RT) showed the predominant of CRF02_AG (n = 104) out of a set of 169 samples correctly sequenced in both regions (PR-RT). It is followed by CRF06_cpx (n = 21), G (n = 5), CRF43_02G (n = 2), CRF37_cpx (n = 2), A1 (n = 1) and URFs (n = 34).

The genetic distribution of the 9 samples amplified only at reverse transcriptase level was as follows: G (n = 1), U/CRF02_AG (n = 1), CRF02_AG (n = 5), CRF06_cpx (n = 1), A3 (n = 1).

**Fig 1:**
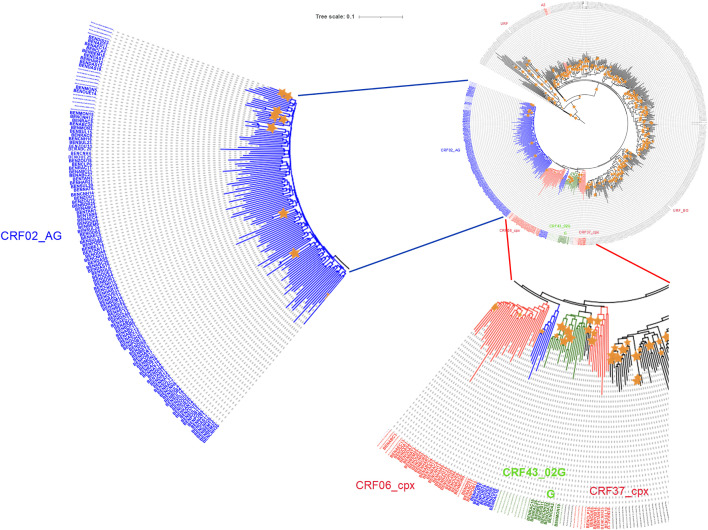
Subtypes diversity of 163 (PROT-RT) sequences. Phylogenetic analysis based on the neighbor-joining method of the HIV-1 (PROT-RT) sequences covering around 1300 bp of the HIV genome. Orange stars indicated bootstraps values greater than 70 out of 100 replicates.

The proximity of the one hundred and sixty-three sequences that generated a unique fragment covering the entire protease and part of the reverse transcriptase was illustrated in (Fig 1).

### Drug resistance mutations associated to NRTIs, NNRTIs and PIs identified in the study

One hundred sixty-four patients carried at least one drug resistance mutations giving overall prevalence with (164/178; 92.1%). (141/178; 79.2%), (160/178; 89.9%) and (7/169; 4.1%) patients carried at least one drug resistance associated to NRTIs, NNRTIs and PIs respectively.

**Fig 2:**
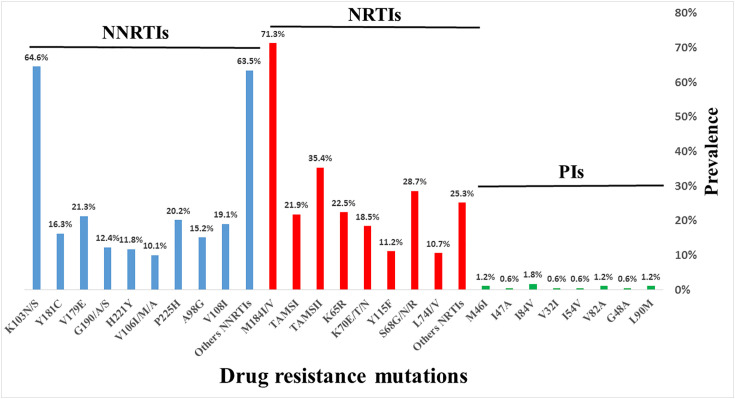
Drug resistance mutations associated to NRTIs, NNRTIs and PIs. NRTIs: nucleoside reverse transcriptase inhibitors, NNRTIs: non-nucleoside reverse transcriptase inhibitors, PIs: Protease inhibitors.

Among NRTIs drug resistance associated mutations, the M184I/V, TAMSI: (M41L, L210W, T215Y) and TAMSII: (D67N, K70R, K219Q/E, T215I/V) represented (71.3%), (21.9%), (35.4%) respectively. Mutations S68G/N/R, K65R, K70E/T/N, Y115F, L74I/V were observed in proportion (28.7%), (22.5%), (18.5%), (11.2%) and (10.7%) respectively. The others NRTIs mutations observed represented (25.3%) (Fig 2).

For NNRTIs, the K103N/S was the most preponderant mutations encountered with a proportion of 64.6% followed by V179E (21.3%), P225H (20.2%), V108I (19.1%), Y181C (16.3%), A98G (15.2%), V106I/M/A (10.1%). The others NNRTIs mutations observed represented 63.5% (Fig 2). The I84V (1.8%) mutation was the major associated PIs encountered followed by L90M, V82A, M46I each encountered twice (1.2%) and I47A, V32I, I54V, G48A, each encountered once (0.6%) (Fig 2).

## Discussion

The aim of this study was to assess the prevalence of virological failure and review genetic diversity and resistance mutations associated with antiretroviral drugs in patients on antiretroviral treatment for at least 12 months before DTG era in Benin.

Benin began treating HIV-infected patients in 2002 through the Benin initiative for access to antiretrovirals (IBAAR) and the first line treatment was composed of AZT/D4T+3TC plus NVP/EFV or non-boosted Indinavir [14].

Already known to be the predominant strain encountered in studies on HIV-1 in the country and in the West African sub-region [9,15–17], the CRF02_AG remained once again the main one identified in this study with the proportion of (109/178; 61.2%) similar to which reported among children in Mali (66.7%) [18]. The others strains circulating with CRF02_AG in the country were CRF06_cpx (22/178; 12.4%) also identified in Mali and Burkina Faso [17–19]. Pure subtypes G (6/178; 3.4%), A3 (1/178; 0.6%), A1 (1/178; 0.6%) were also identified in Ghana [19].

The CRF43_02G (2/178; 1.1%) has been reported at a proportion of 16% in Nigeria [20]. It presence in Benin is becoming more evident, as it has recently been reported in children in the country [16]. The fluid mobility of the Beninese and Nigerian populations across the border that separates the two countries could easily explained the presence of this new recombinant. It’s the first time that CRF37_cpx has been isolated in Benin. It is one of the strains contributing to the high genetic diversity of HIV-1 in Central Africa where a proportion of 2.3% has been reported [21]. Its presence in West Africa could be explained by the migration of the population from Central Africa to West Africa. The proportion of CRF37_cpx reported in our study could mean that its introduction into the country was not long ago or that the genetic recombinations contributing to it genesis were recent. Indeed, the strains involved in its composition CRF01_AE, CRF02_AG, A, G, U have been reported in the country in the previous studies [15,16].

The isolated strains were known to be involved in genetic recombination during coinfections or superinfections [22–24], generating news CRFs or URFs. In our study, some unique recombinant forms (URFs) were reported. Similar proportion (17.8%) has been reported in China [25].

There was clear evidence that combination antiretroviral therapy inhibits viral replication [26] and achieves HIV-1 viral load suppression [27]. In this situation, therapeutic adherence was of capital importance [28] especially in the context where antiretroviral treatment is lifelong [29]. This is why efforts were being made by national HIV control programs in conjunction with technical and financial partners through projects. The most recent was the USAID PEPFAR project, which has invested heavily in training those involved in the care of people living with HIV, from screening for infection to achieving undetectable viral load in patients on treatment [30]. However, it was clear that the difficulties inherent in providing care persist [31]. One of them was the lack of treatment adherence which was the first factor responsible for therapeutic failure [29]. In the case of our study, a virological failure proportion of 48.37% was reported. In addition to non-adherence to treatment, which could explain this proportion of virological failure, forgetfulness and side effects linked to taking the tablets and stigma could also be elements that reinforce this observation. Variable proportions have been reported in Africa such as West Africa in Togo (19.6%) [32], North Africa as Morocco (18.20%) [33], East Africa such as Rwanda and Tanzania [34,35], Central Africa as Cameroon (25.5%) [36], Malawian among children (66%) [37], South Africa (14.7%) among women living with HIV [38].

Virological failure means an increase in viremia. In this context, the virus enhances it replication by making amino acid substitutions [39] along its genome based on the antiretroviral drugs to which it has been exposed. This situation was known as a resistance mutation [40]. In our study, 92.1% (164/178) of patients in virological failure carried at least one drug resistance mutation. These high proportion is also observed for NRTIs and NNRTIs resistance mutations respectively at least (141/178; 79.2%) and (160/178; 89.9%) resistance mutations. Indeed, discontinuities in the daily intake of tablets [41] could be observed in these patients, which would result in low plasma concentrations of antiretrovirals [41], thus providing a favourable environment for the virus to carry out selections for resistance mutations. In our study, RT amino acid substitutions in 184 position has been observed at (127/178; 71.3%). This mutation has been reported in studies conducted in Benin [14] and recently among children [16]. It was reported in the study on transmitted HIV-1 resistance in Benin [15]. Some similar results have been reported in Africa such as Senegal [42], Ivory Coast [43], and Mali [44], in Europe such as France [45], and in Asia, such as Taïwan [46,47].

The benefits of combination antiretroviral drugs were recognized early on for their increased effectiveness against HIV [29]. In this regard, AZT or D4T were combined with 3TC or FTC [29]. It then becomes clear that, over such a long period under these molecules to which the patient did not adhere, the virus selects resistance mutations. In our study, TAMSI and TAMSII have been observed at (21.9%) and (35.4%) respectively. These proportions, which appear to be low, could be easily explained by the low number of patients in our study under this line but also by the transition of certain patients under the TDF/ABC + 3TC + EFV line. Similar proportions have been reported in Uganda (1% to 20%) [48] and Thailand (43% to 90%) [47].

In addition to doubling up on NRTIs, NNRTIs or PIs have been added to the treatment regimen with the same goal of ensuring good viral inhibition and reducing the number of deaths related to the disease [49]. NVP and EFV were the NNRTIs combined to AZT/D4T + 3TC/FTC. The predominant drug resistance mutation associated to these drugs was K103N/S (64.6%) in our study. This proportion is not surprising because it has been reported that even at low frequency, K103N would significantly increase the risk of virological failure in patients on NVP and EFV [50]. It is has been reported in patients under a PI-based regimen who previously failed under NNRTIs [51]. It can persist for up to five years in a pregnant woman who has received a single dose of NVP to prevent mother-to-child transmission of HIV-1 [52].

From the beginning of the treatment of HIV-positive people in Benin in 2002, Indinavir was the PI associated with the AZT/D4T+3TC/FTC combination [14], then it was replaced by EFV, and today, it is used as a second line for patients who have failed first-line treatment [39]. This could explain the low proportion of resistance mutations associated with PIs reported in our study. Among these mutations, the major encountered was I84V (1.8%) followed by L90M, V82A and M46I each identified twice (1.2%). The L90M has been reported recently among children in Benin [16]. The I84V and V82A have been reported among adults in Gabon [53].

### Limitations

Our study was not limitations. The sample size was small considering the large number of patients on ART in Benin. The criteria used to select patients for inclusion could be one of the factors contributing to the introduction of bias that would have reduced the sample size.

## Conclusion

The study shows a high genetic diversity with the presence of new strains and a high prevalence of resistance mutations associated. This underlines the need to regularly review data on genetic diversity and resistance among patients receiving antiretroviral therapy in the country.

## Supporting information

S1 File Genbank Accession Numbers. (DOCX)
