# Supplementary material for: HIV-1 genetic diversity and reverse transcriptase resistance mutations in Benin before dolutegravir era, West Africa
Source: PLoS One. 2026 May 21;21(5):e0348800. doi: 10.1371/journal.pone.0348800 (PMC13193392; doi:10.1371/journal.pone.0348800)
Supplement: S1 File — (DOCX) [file pone.0348800.s001.docx]

BankIt2940802 BENSUL4 PV382981

BankIt2940802 BENSUL5 PV382982

BankIt2940802 BENSUL6 PV382983

BankIt2940802 BENSUL10 PV382984

BankIt2940802 BENSUL11 PV382985

BankIt2940802 BENSUL12 PV382986

BankIt2940802 BENSUL13 PV382987

BankIt2940802 BENSUL15 PV382988

BankIt2940802 BENSUL18 PV382989

BankIt2940802 BENSUL20 PV382990

BankIt2940802 BENSUL22 PV382991

BankIt2940802 BENSUL23 PV382992

BankIt2940802 BENSUL24 PV382993

BankIt2940802 BENSUL26 PV382994

BankIt2940802 BENBET1 PV382995

BankIt2940802 BENBET5 PV382996

BankIt2940802 BENBET6 PV382997

BankIt2940802 BENBET14 PV382998

BankIt2940802 BENBET21PROT PV382999

BankIt2940802 BENBET21RT PV383000

BankIt2940802 BENDIS3 PV383001

BankIt2940802 BENDIS8 PV383002

BankIt2940802 BENDIS9 PV383003

BankIt2940802 BENDIS11 PV383004

BankIt2940802 BENDIS12 PV383005

BankIt2940802 BENDIS16 PV383006

BankIt2940802 BENDIS17RT PV383007

BankIt2940802 BENDIS19PROT PV383008

BankIt2940802 BENDIS19RT PV383009

BankIt2940802 BENDIS23 PV383010

BankIt2940802 BENAEC1 PV383011

BankIt2940802 BENAEC4 PV383012

BankIt2940802 BENAEC5 PV383013

BankIt2940802 BENAEC9 PV383014

BankIt2940802 BENAEC11 PV383015

BankIt2940802 BENAEC12 PV383016

BankIt2940802 BENAEC13 PV383017

BankIt2940802 BENAEC15 PV383018

BankIt2940802 BENRAC5 PV383019

BankIt2940802 BENRAC6 PV383020

BankIt2940802 BENRAC7 PV383021

BankIt2940802 BENRAC8 PV383022

BankIt2940802 BENRAC9 PV383023

BankIt2940802 BENRAC10 PV383024

BankIt2940802 BENRAC11 PV383025

BankIt2940802 BENRAC12 PV383026

BankIt2940802 BENRAC13 PV383027

BankIt2940802 BENRAC16 PV383028

BankIt2940802 BENRAC17 PV383029

BankIt2940802 BENRAC19 PV383030

BankIt2940802 BENRAC22 PV383031

BankIt2940802 BENRAC26 PV383032

BankIt2940802 BENCNH2 PV383033

BankIt2940802 BENCNH6 PV383034

BankIt2940802 BENCNH8 PV383035

BankIt2940802 BENCNH10 PV383036

BankIt2940802 BENCNH12 PV383037

BankIt2940802 BENCNH13 PV383038

BankIt2940802 BENCNH14 PV383039

BankIt2940802 BENCNH15 PV383040

BankIt2940802 BENCNH16 PV383041

BankIt2940802 BENCNH20 PV383042

BankIt2940802 BENCNH22 PV383043

BankIt2940802 BENCNH25 PV383044

BankIt2940802 BENCNH30 PV383045

BankIt2940802 BENCNH31 PV383046

BankIt2940802 BENABC3 PV383047

BankIt2940802 BENABC4 PV383048

BankIt2940802 BENABC9 PV383049

BankIt2940802 BENABC11 PV383050

BankIt2940802 BENABC13 PV383051

BankIt2940802 BENABC17 PV383052

BankIt2940802 BENABC21 PV383053

BankIt2940802 BENABC23 PV383054

BankIt2940802 BENABC24 PV383055

BankIt2940802 BENABC26 PV383056

BankIt2940802 BENABC27 PV383057

BankIt2940802 BENABC28 PV383058

BankIt2940802 BENABC29 PV383059

BankIt2940802 BENMON2 PV383060

BankIt2940802 BENMON3 PV383061

BankIt2940802 BENMON5 PV383062

BankIt2940802 BENMON9 PV383063

BankIt2940802 BENMON10RT PV383064

BankIt2940802 BENMON15 PV383065

BankIt2940802 BENMON19 PV383066

BankIt2940802 BENZOU1 PV383067

BankIt2940802 BENZOU2 PV383068

BankIt2940802 BENZOU3 PV383069

BankIt2940802 BENZOU5 PV383070

BankIt2940802 BENZOU7 PV383071

BankIt2940802 BENZOU8 PV383072

BankIt2940802 BENZOU11 PV383073

BankIt2940802 BENZOU15 PV383074

BankIt2940802 BENZOU18 PV383075

BankIt2940802 BENDAS1 PV383076

BankIt2940802 BENDAS4 PV383077

BankIt2940802 BENDAS6 PV383078

BankIt2940802 BENDAS7 PV383079

BankIt2940802 BENDAS8 PV383080

BankIt2940802 BENDAS9 PV383081

BankIt2940802 BENDAS12 PV383082

BankIt2940802 BENDAS15 PV383083

BankIt2940802 BENDAS17 PV383084

BankIt2940802 BENDAS19 PV383085

BankIt2940802 BENCLP2 PV383086

BankIt2940802 BENCLP6 PV383087

BankIt2940802 BENCLP10 PV383088

BankIt2940802 BENOUE3 PV383089

BankIt2940802 BENOUE4 PV383090

BankIt2940802 BENOUE6 PV383091

BankIt2940802 BENOUE8 PV383092

BankIt2940802 BENOUE9 PV383093

BankIt2940802 BENOUE14 PV383094

BankIt2940802 BENOUE20 PV383095

BankIt2940802 BENOUE21 PV383096

BankIt2940802 BENOUE25 PV383097

BankIt2940802 BENGOH5 PV383098

BankIt2940802 BENGOH20 PV383099

BankIt2940802 BENGOH21PROT PV383100

BankIt2940802 BENGOH21RT PV383101

BankIt2940802 BENGOH26 PV383102

BankIt2940802 BENPOB2 PV383103

BankIt2940802 BENPOB5 PV383104

BankIt2940802 BENPOB6 PV383105

BankIt2940802 BENPOB11 PV383106

BankIt2940802 BENPOB12 PV383107

BankIt2940802 BENPOB13 PV383108

BankIt2940802 BENPOB14 PV383109

BankIt2940802 BENPOB17 PV383110

BankIt2940802 BENPOB19 PV383111

BankIt2940802 BENODA1 PV383112

BankIt2940802 BENODA2 PV383113

BankIt2940802 BENODA4 PV383114

BankIt2940802 BENODA5 PV383115

BankIt2940802 BENODA7 PV383116

BankIt2940802 BENODA9 PV383117

BankIt2940802 BENODA10 PV383118

BankIt2940802 BENNAT1 PV383119

BankIt2940802 BENNAT3 PV383120

BankIt2940802 BENNAT4 PV383121

BankIt2940802 BENNAT6 PV383122

BankIt2940802 BENNAT14 PV383123

BankIt2940802 BENNAT27RT PV383124

BankIt2940802 BENBEM1PROT PV383125

BankIt2940802 BENBEM1RT PV383126

BankIt2940802 BENBEM5RT PV383127

BankIt2940802 BENBEM7RT PV383128

BankIt2940802 BENBEM9 PV383129

BankIt2940802 BENBEM12 PV383130

BankIt2940802 BENBEM14 PV383131

BankIt2940802 BENBEM18 PV383132

BankIt2940802 BENBEM22 PV383133

BankIt2940802 BENBEM23PROT PV383134

BankIt2940802 BENBEM23RT PV383135

BankIt2940802 BENBEM25 PV383136

BankIt2940802 BENKAN4 PV383137

BankIt2940802 BENKAN14 PV383138

BankIt2940802 BENKAN17RT PV383139

BankIt2940802 BENKAN25RT PV383140

BankIt2940802 BENBOR1 PV383141

BankIt2940802 BENBOR4 PV383142

BankIt2940802 BENBOR5 PV383143

BankIt2940802 BENBOR6 PV383144

BankIt2940802 BENBOR25 PV383145

BankIt2940802 BENTAN1 PV383146

BankIt2940802 BENTAN3 PV383147

BankIt2940802 BENTAN8 PV383148

BankIt2940802 BENTAN10 PV383149

BankIt2940802 BENTAN11 PV383150

BankIt2940802 BENTAN12PROT PV383151

BankIt2940802 BENTAN12RT PV383152

BankIt2940802 BENTAN13 PV383153

BankIt2940802 BENTAN17 PV383154

BankIt2940802 BENTAN23RT PV383155

BankIt2940802 BENDJG14 PV383156

BankIt2940802 BENHAR1 PV383157

BankIt2940802 BENHAR6RT PV383158

BankIt2940802 BENHAR7 PV383159

BankIt2940802 BENHAR8 PV383160

BankIt2940802 BENHAR12 PV383161

BankIt2940802 BENHAR16 PV383162

BankIt2940802 BENHAR21 PV383163

BankIt2940802 BENHAR27 PV383164
